# Tight stomatal regulation and high intrinsic capacity of carbon assimilation inferred from isotopic composition negatively correlate with drought-driven decline in sclerophyllous species

**DOI:** 10.1093/aob/mcag100

**Published:** 2026-04-24

**Authors:** Frida I Piper, Susana Paula, Rocío Urrutia-Jalabert

**Affiliations:** Instituto de Ciencias Biológicas, Universidad de Talca, campus Lircay, Av. Lircay SN, Talca 3460000, Chile; Instituto de Ecología y Biodiversidad, Universidad de Chile, Las Palmeras 3425, Santiago 7800003, Chile; Núcleo Milenio Límite de la Vida en Patagonia (Lili), Universidad Austral de Chile, Valdivia 5090000, Chile; Instituto de Ecología y Biodiversidad, Universidad de Chile, Las Palmeras 3425, Santiago 7800003, Chile; Instituto de Ciencias Ambientales y Evolutivas, Universidad Austral de Chile, Campus Isla Teja, Valdivia 509000, Chile; Núcleo Milenio Límite de la Vida en Patagonia (Lili), Universidad Austral de Chile, Valdivia 5090000, Chile; Departamento de Ciencias Forestales, Facultad de Ciencias Agropecuarias y Medioambiente Universidad de la Frontera, Temuco 4780000, Chile; Instituto de Conservación, Biodiversidad y Territorio, Facultad de Ciencias Forestales y Recursos Naturales, Universidad Austral de Chile, Campus Isla Teja, Valdivia 5090000, Chile; Centro de Ciencia del Clima y la Resiliencia, CR2, Universidad de Chile, Santiago 7800003, Chile

**Keywords:** Angiosperms, carbon starvation, browning, dehydration, forest decline, megadrought, osmoregulation, tree growth, tree mortality

## Abstract

**Background and Aims:**

Long-term droughts reduce the carbon (C) and water pools of trees, causing mortality. However, the underlying physiological mechanisms explaining tree mortality remain unclear, and evidence from mature forests is scarce. The Chilean sclerophyllous forest has shown an abrupt and severe decline after >10 years of continuous drought, evidenced by browning and defoliation. We hypothesized that limited photosynthesis led to reduced concentrations of non-structural carbohydrates (NSCs) and consequently impaired dehydration tolerance, and that drought resistance imposed a penalty in growth rates.

**Methods:**

We quantified the species-specific level of crown decline in 11 woody species and sampled unhealthy and healthy individuals within species to measure isotopic composition (δ^18^O and δ^13^C) and NSC concentrations in twigs and leaves formed in 2019 and 2020 as well as mean basal area increment (BAI) before (1999–2009) and during the megadrought (2010–2020). The effects of species-specific crown decline and intraspecific health status on isotopes, NSCs and BAI were evaluated using linear mixed-effects models.

**Key Results:**

δ^13^C decreased with species’ crown decline, while δ^18^O did not change, indicating biochemical limitations to C assimilation in species with higher decline. Stomatal limitations to C assimilation, inferred from higher δ^18^O, along with lower NSC and sugar concentrations, were found in healthy individuals of species with less crown decline and in unhealthy individuals of species with higher crown decline. Unhealthy individuals grew less than healthy ones in both periods examined and regardless of species’ crown decline.

**Conclusions:**

Species’ crown decline in the sclerophyllous forest of Central Chile was associated with biochemical and diffusive limitations on photosynthesis, but it was not related to reduced levels of NSCs or to historical high or low growth rates. The results suggest that a timely stomatal regulation successfully prevents dehydration and reduces crown decline only when it occurs along with a high capacity of C assimilation.

## INTRODUCTION

Forests have the potential to mitigate climate change by converting anthropogenically increased atmospheric CO_2_ emissions into biomass. However, this potential is reduced by tree decline and mortality, processes that have been affecting forests in diverse regions of the world, mainly due to climate change-driven droughts and heatwaves (van Mantgem *et al*., 2009; Allen *et al*., 2015; Greenwood *et al*., 2017; Neumann *et al*., 2017; Andrus *et al*., 2024). Our current capacity to predict forest responses to these climatic factors is, however, still limited by an incomplete understanding of the causal physiological mechanisms (McDowell *et al*., 2008, 2022; Adams *et al*., 2017; Körner, 2019). In particular, there is a gap between the cumulative experimental evidence and the mechanisms that operate in the field, where the droughts can have a long duration and interact with other dynamic environmental variables, making their simulation challenging (Gessler *et al*., 2018; Peltier *et al*., 2023). Field data of trees exposed to natural long-term droughts are therefore necessary to validate physiological theories about causal mechanisms of tree decline and mortality.

A limited carbon (C) supply may impair the maintenance of metabolic, defensive or hydraulic functions, a process defined as C starvation (McDowell *et al*., 2022). In trees subjected to long-term and severe droughts (i.e. reduction in precipitation relative to historical means), the demand for C should ultimately exceed the gain leading to reductions in starch or total non-structural carbohydrates (NSCs) (Peltier *et al*., 2023). However, many drought experiments, especially those carried out with seedlings and saplings, show that NSC concentrations do not always decrease in response to droughts (e.g. Adams *et al*., 2017). It has also been highlighted that there is a need to account for data from mature trees, which in contrast to saplings may require longer droughts to reduce their NSC concentrations, since their NSC pools are larger (Gessler *et al*., 2018). In the same line, it has been proposed that C starvation perhaps only begins once trees have exceeded some threshold in drought duration (Peltier *et al*., 2023), so the little support for this mechanism could be because drought was not prolonged enough to substantially impact NSC pools (Hoch *et al*., 2003; Würth *et al*., 2005). Consistent with this framework, it has been reported that trees of *Pinus edulis* significantly reduced sapwood starch concentrations after a decade of 45 % reduction in precipitation (Peltier *et al*., 2023).

Reduction in the availability of NSCs may compromise the capacity of trees to resist droughts. Two major strategies of drought resistance, i.e. drought avoidance and dehydration tolerance, depend on the tree C status (McDowell *et al*., 2022). The most universal drought avoidance response, i.e. stomatal closure, forces trees to survive at the expense of their C reserves (McDowell *et al*., 2022). Likewise, dehydration tolerance is globally explained by the capacity of plants to accumulate compatible solutes that maintain the turgor of tissues (Bartlett *et al*., 2012). Soluble carbohydrates can act themselves as osmolytes or be precursors of other well-known osmotically active molecules such as proline and glycine-betaine (Martínez-Vilalta *et al*., 2016). Thus, the accumulation of soluble carbohydrates correlates with the capacity for osmotic adjustment and drought tolerance (Arndt *et al*., 2001). Sugar accumulation under dry conditions may be preferentially supported from the ongoing photoassimilation (Piper *et al*., 2017), but it can also be supported from storage remobilization when photosynthesis is low (Martínez-Vilalta *et al*., 2016). Osmoregulation is commonly impaired in experimentally C-limited plants (O’Brien *et al*., 2014; Sevanto *et al*., 2014; Sapes *et al*., 2021). This impairment can emerge from drought-induced limited phloem functioning, which can restrict the long-distance transport of osmolytes or of their precursors (Sevanto *et al*., 2014; Savage *et al*., 2016; Sevanto, 2018).

Whether long-term droughts can cause tree decline via a fail to meet osmotic requirements remains unknown. However, we could expect this possibility in those ecosystems where arid conditions determine high osmotic demands, such as Mediterranean ecosystems (Bartlett *et al*., 2012). A global meta-analysis showed that Mediterranean biomes are the only ones where plants exhibit a remarkable across-organ sugar accumulation at the end of summer (Martínez-Vilalta *et al*., 2016). Accordingly, Bartlett *et al*. (2012) showed that the lowest osmotic potential values at full turgor and at the turgor loss point worldwide are found in arid biomes such as Mediterranean ones. Given the high osmotic requirements in Mediterranean vegetation, it is worth asking whether a pervasive drought limits the C availability for osmoregulation in Mediterranean forests.

Different mechanisms of drought resistance may impose a growth penalty. While reduced growth in drought-avoider species has been proposed to be a direct consequence of the deterioration of the C balance and the hydraulic performance under droughts (Jansen *et al*., 2013; Cailleret *et al*., 2017), it may also be an active strategy to reallocate C from growth to other sinks such as storage and/or osmoregulation (Wiley *et al*., 2013, 2017; Dietze *et al*., 2014; Piper *et al*., 2015). Growth is more sensitive to drought than photosynthesis, which should lead to increasing levels of C reserves under moderate droughts (Boyer, 1970; Muller *et al*., 2011). However, under long-lasting severe droughts, reduced C availability could limit the requirements for turgor and cell-wall thickening, two key processes of xylogenesis (Buttò *et al*., 2025). Recent studies have also found that growth and photosynthesis may be reduced in parallel under drought (Thompson *et al*., 2023; Reyes-Bahamonde *et al*., 2026). Yet, observations of dying trees do not show consistent results, with growth patterns of dying trees varying among different studies and species. In some cases, the trees that died from drought were those with low growth sensitivity to water availability (Herrero *et al*., 2023). In contrast, growth suppression for various years before mortality is a common feature of trees that suffer dieback (Cailleret *et al*., 2017; Colangelo *et al*., 2017; Camarero *et al*., 2018; Castellaneta *et al*., 2022), suggesting that different factors may predispose trees to drought-induced mortality.

**Fig. 1.**
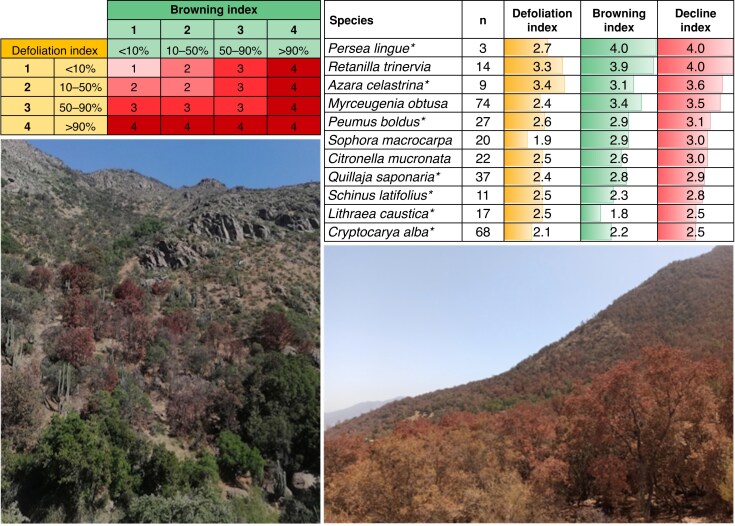
Sclerophyllous evergreen forest showing decline after 10 years of megadrought in La Campana National Park, Central Chile, February 2020. Table at upper left shows the decline level (red area) based on categories (1–4) of defoliation (yellowish area) and browning (green area) representing percentage ranges of the variables. Table at upper right shows the list of studied species (those used for the growth analyses are marked with an asterisk), and the average defoliation, browning and decline values recorded across a 1-km-long trail. The number of individuals used to assess the decline level is indicated (*n*). Photo credits: Susana Paula

In the period 2010–2022, central and southern Chile experienced the longest-term drought in the history of the region (i.e. megadrought), and an extreme hyperdrought in 2019 (Garreaud *et al*., 2017, 2020, 2025). Annual precipitation during the megadrought was *c*. 35 % below the historical average, being the lowest precipitation recorded since 1920 in Central Chile (Garreaud *et al*., 2025). The megadrought occurred on top of a precipitation reduction reported between 1960 and 2020 across the country (Boisier *et al*., 2025). Dry conditions during the megadrought were unprecedented in the recent history of all Mediterranean-type ecosystems (Miranda *et al*., 2023). The sclerophyllous forest responded to this sustained and acute drought with a large-scale, abrupt browning and productivity decline affecting >90 % of the forest in <100 d between 2019 and 2020, although the level of decline varied interspecifically (Miranda *et al*., 2023) (Fig. 1). Here we studied the potential physiological mechanisms underlying this forest decline by conducting a series of intraspecific comparisons between healthy and unhealthy individuals across 11 species exhibiting different levels of decline (as a proxy of drought sensitivity). To this end, we measured NSC concentrations in leaves and twigs, as well as the isotopic enrichment of twigs in ^18^O and ^13^C to distinguish between possible physiological effects of drought on stomatal conductance and biochemical efficiency (Scheidegger *et al*., 2000). Finally, we also measured tree-ring growth to explore relationships between drought-driven decline and growth rates. We hypothesized that (1) limited photosynthesis led to reduced total NSC concentrations and particularly sugars, impairing drought tolerance, and (2) reduced drought-driven decline imposes a penalty in growth rates. We anticipated that, within species, total NSC and sugar concentrations were lower in unhealthy than in healthy individuals, particularly for species exhibiting higher levels of decline. These decreases are expected to result from limited photosynthesis, due to reduced stomatal conductance or to biochemical constraints driven by drought. As evidence of reduced stomatal conductance limiting photosynthesis, we expect increasing δ^13^C and δ^18^O, while as evidence of biochemical constraints limiting photosynthesis, we expect decreasing δ^13^C along with invariable or increasing δ^18^O (Scheidegger *et al*., 2000). We further expected lower growth in healthy than in unhealthy individuals, particularly in the species with lower crown decline, reflecting a trade-off between growth and drought resistance.

## MATERIALS AND METHODS

### Study area and species

We worked in La Campana National Park, in the Valparaíso region of Chile, specifically in an area located on a south-facing slope called Granizo (32°58′S, 71°07′W, 430 m above sea level). The climate in this area is Mediterranean, with a mean annual temperature of 14.3 °C and mean annual precipitation of 435 mm, with negligible precipitation (*c*. <3 mm per month) between November and March (Quillota climatic station, located *c*. 16 km away) (Luebert and Pliscoff, 2006). For the warmest (December–February) and coldest (June–August) months, mean temperatures are 18.5 and 10.2 °C, respectively (Luebert and Pliscoff, 2006). Within the park itself, climatic conditions become cooler and wetter with increasing elevation. On the seaward slopes of the mountains, fogs are relatively frequent (Rundel and Weisser, 1975). Sclerophyllous forests dominate south-facing, low to moderate slopes (Rundel and Weisser, 1975). Several tree species, all angiosperms and mostly sclerophyllous evergreen, can be found co-occurring (Rundel and Weisser, 1975), the dominant species being *Cryptocarya alba* (peumo), *Beilschmiedia miersii* (belloto del norte), *Peumus boldus* (boldo) and *Azara celastrina* (lilén) (Rundel and Weisser, 1975) (Fig. 1).

### Sampling

Since 2010, precipitation in the area has been *c.* 25–45 % below normal every year (Quebrada Alvarado, Dirección General de Aguas). In summer 2020, i.e. 10 years after the climatic anomalies started, in what was so-called the megadrought, and the hyperdrought of 2019, forest browning was massively evident at the landscape scale (Miranda *et al*., 2023). However, there was high interspecific variability in the type and severity of the responses. Some species responded through browning, but others responded via defoliation, and some others exhibited a mix of both responses. To account for the different types of responses, we created an index of decline that combined the level of defoliation and the level of browning by converting ranges (as percentages) of defoliation or browning to categorical levels ranging from 1 to 4. Our index therefore combined the two most typical tree decline responses in a single value of decline as a proxy for species-level drought sensitivity (Fig. 1). This approach is more adequate for interspecific comparisons than the typically used defoliation percentage or browning percentage, as different species may possess different types of decline responses, which our index allows for comparison. We quantified the degree of decline of all woody species along a circular trail called ‘La Canasta’, most of which were trees. The trail is about 1 km long and crosses a typical Mediterranean sclerophyllous forest, with some representative areas of hygrophilous forest. Every 10 m, we identified the nearest tree (at least 5 cm in diameter at breast height) and shrub (at least 1.5 m in height) on each side of the trail. For each of these plants, we estimated the degree of decline as described above. Using this procedure, we recorded only three individuals of *Persea lingue* (one of the studied species; see below), all of them with the highest level of decline. However, consistent with the level of decline estimated for this species, most of the *Persea lingue* trees observed in the transect were also completely browned.

Sampling was carried out at the end of February 2020, when the decline of this forest was still in progress (Miranda *et al*., 2023). We sampled 11 species based on two criteria. First, the whole set of species should cover as much variation in decline levels as possible. Therefore, we selected species to represent from low to severe decline levels, according to the mean decline index recorded for each species across the trail (Fig. 1). The second criterion for species selection was the availability of healthy and unhealthy trees for a given species. Thus, species for which healthy individuals, defined by <10 % defoliation and <10 % browning, were absent in the study area, were excluded from our analysis. Unhealthy individuals were defined as living trees with >50 % browning and/or >50 % defoliation. For each selected species (Fig. 1), we sampled five healthy and five unhealthy individuals, which were intermingled and sparse in an area of 21 ha (including the 1-km trial used for decline assessment), and separated from each other by 5–15 m. This rather low sample size is explained by the scarce availability of healthy individuals in the species with higher decline. The average decline level of the sampled unhealthy trees did not correlate with the species-specific decay level recorded across the 1-km trail (Pearson’s correlation = 0.52, *P* = 0.103).

For NSC and isotope analyses, we cut one 70-cm-long terminal branch with fully expanded, sun-exposed leaves per tree; we kept each branch in a plastic bag in cool conditions until arriving at the field laboratory. Tissue collection was conducted between 1000 and 1300 h. In the laboratory, twigs corresponding to the current year (spring 2019 to summer 2020, hereafter ‘2020’) and previous year (spring 2018 to summer 2019, hereafter ‘2019’) were separated using the presence of scars to differentiate them and defoliated. In unhealthy trees of some species, leaves fell off very easily, so it was not possible to differentiate the different cohorts of leaves. Therefore, leaves from 2019 and 2020 were pooled in both healthy and unhealthy trees. Twig and leaf samples were then dried in a forced-air stove (Memmert GmbH) at 70 °C for 48 h, after which they were ground into a fine powder and stored over silica gel at 4 °C until NSC determinations and isotopic analyses were conducted.

To evaluate tree growth, the diameter at breast height (DBH, 1.35 m) was measured and two stem cores were collected per tree at breast height, using a 5.15-mm-wide increment borer (Haglöf), in February 2022. To avoid causing damage to trees/shrubs, only eight species were sampled due to their adequate stem size for core boring: *Azara celastrina*, *Cryptocarya alba*, *Lithraea caustica*, *Myrceugenia obtusa*, *Persea lingue*, *Peumus boldus*, *Quillaja Saponaria* and *Schinus latifolius*. In several Chilean sclerophyllous species, including some of the ones sampled in this study, tree-ring formation has been reported and tree-ring width chronologies have been successfully developed (Venegas-González *et al*., 2023; Gibson-Carpintero *et al*., 2024; González-Reyes *et al*., 2024; Gipoulou-Zúñiga *et al*., 2025). The sampled species represent different levels of decline (Fig. 1). At least ten healthy and ten unhealthy individuals, including the trees that were sampled for physiological measurements, were sampled. All stem cores were placed in plastic straws and labelled for growth analyses.

### Measurements

*Carbon and oxygen isotope analyses.* To help elucidate if trees ultimately adjust their stomatal behaviour and C assimilation during periods of drought, we determined the wood isotopic composition (δ^13^C and δ^18^O) of twigs that were formed during the occurrence of drought, specifically in 2019 and 2020. Carbon and oxygen isotope analyses were conducted at the Laboratorio de Investigación en Suelos, Aguas y Bosques, University of Concepción, Chile. Samples of 250 μg for δ^13^C (300 μg for δ^18^O) were weighed on a microbalance (ME36S, Sartorius) and then placed in tin (silver for δ^18^O) foil capsules and converted to CO_2_ (CO for δ^18^O) using an NC 2500 (Carlo Erba) elemental analyser (HT pyrolysis oven, Hekatech for δ^18^O) interfaced with a Finnigan DeltaPlus (Delta V Advantage for δ^18^O) isotope ratio mass spectrometer (Thermo Fisher Scientific). Stable isotope ratios were expressed as per mil deviations using the δ notation relative to Vienna Pee Dee Belemnite (VPDB) for C and Standard Mean Ocean Water (SMOW) for O. The standard deviation for repeated analyses was better than 0.2 ‰ for δ^13^C and δ^18^O.

*NSC determination.* We determined total NSC (hereafter NSC) concentrations as the sum of the three most abundant low-molecular-weight sugars (sucrose, glucose and fructose, SGF) and starch, based on Hoch *et al*. (2002) and Wong *et al*. (2003). About 13 mg of dried powder from twig and leaf samples was extracted with 1.6 mL distilled water at 100 °C for 60 min. An aliquot of the extract was used to determine low-molecular-weight soluble sugars after the enzymatic conversion of sucrose and fructose to glucose (invertase and phosphoglucose isomerase from *Saccharomyces cerevisiae*, Sigma Aldrich I4504 and P5381, respectively). The concentration of free glucose was determined photometrically after the enzymatic conversion of glucose to glucose-6-phosphate (Glucose Assay Reagent, G3293 Sigma Aldrich) on a 96-well multiplate reader. Following the degradation of starch to glucose using a purified fungal amylase (‘amiloglucosydase’ from *Aspergillus niger*, Sigma Aldrich 10115) at 45 °C overnight, NSC concentrations were determined in a separate analysis. The starch concentration was calculated as NSCs minus the sum of free sugars (SGF). Total soluble sugar, starch and NSC concentrations were computed on a dry matter basis (%).

*Tree growth determination.* Cores were mounted and sanded, and tree rings were visually dated using a binocular microscope. Tree-ring widths were measured to 0.001-mm precision with a measuring stage (Velmex, Bloomfield NY, USA) interfaced with a computer. Cross-dating accuracy was verified and improved using the program COFECHA (Holmes 1983). Except for *Myrceugenia obtusa*, all species were successfully cross-dated. Tree-ring width chronologies per species were developed using healthy and unhealthy trees and, later, tree-ring series from both categories were separated to assess their differences.

Basal area increment (BAI) has been reported to more realistically represent patterns of forest decline than tree-ring width (Rodríguez-Catón *et al*., 2015). Therefore, the annual BAI of healthy and unhealthy trees per species was calculated for each individual tree-ring width series as:


$$\mathrm{BA}I_{t}=\pi (SR_{t}^{2}\text{–}SR_{t-1}^{2})$$

where SR is the stem radius and *t* is the year of tree ring formation. BAI was then averaged per tree for the period when the megadrought occurred (2010–2020, BAI_2010–2020_) and the previous 10 years (1999–2009, BAI_1999–2009_).

### Statistical analyses

To assess whether isotopic composition, NSC (and starch and SGF) concentrations and radial growth were related to the species’ decline level and to within-species’ tree health status, we conducted a series of linear mixed models (LMMs) in which the response variables were isotopic composition (δ^18^O and δ^13^C, separately for 2019 and 2020 twigs) and the NSC concentration (total NSC, starch and SGF, separately for 2019 and 2020 twigs and pooled for leaves) using the nlme package v.3.1-162 (Pinheiro *et al*., 2016). The species-specific decline level (as a continuous variable, Fig. 1), the health status of each individual (healthy, unhealthy) and their interaction were included as fixed factors in the model, whereas species identity was included as a random factor to account for non-independence among individuals of the same species. For each response variable, we fitted three models using relative maximum likelihood (Bolker *et al*., 2009): a full model (i.e. with the fixed and random factors), a simpler model (with decline level and random factor) and a model with the random term only using the Akaike Information Criterion (AIC) corrected for small sample size (AICc). Note that if interspecific differences in decline level are mediated by differential adjustments in gas exchange and NSC concentrations, an interaction between decline level and health status is expected. For each response variable, the model with lowest AIC (>2 units lower than the other model) was considered the most robust, and for this model the statistical significance of the fixed part of the model was evaluated through ANOVA. For statistically significant factors, post-hoc multiple comparisons (Tukey’s procedure) were conducted using the emmeans package 1.10.2 (Hothorn *et al*., 2008). To reveal the potential influences of outliers and extreme values, the fit of the models was evaluated with a graphical examination of the residuals and fitted values. These analyses were performed in R 4.2.3.

LMMs were also used to analyse radial growth (BAI_1999–2009_ and BAI_2010–2020_). These models had a similar structure to those used for isotopes and NSC concentrations, but they also included tree age or DBH as a covariables. We further examined tree growth differences during the pre-megadrought (1999–2009) and megadrought (2010–2020) periods, and tree size differences (as DBH) between healthy and unhealthy individuals per species using the Wilcoxon rank-sum test (Hentschel *et al*., 2014). This test is robust under non-standard distributions and temporal autocorrelation (Gibbons and Chakraborti, 2011).

## RESULTS

### Decline level among species

A total of 302 individuals were assessed for defoliation and browning (Fig. 1). The resulting index of decline ranged from 4 to 2.5, corresponding to the most severely affected species (*Retanilla* and *Persea*) and the least affected species (*Lithraea* and *Cryptocarya*), respectively (Fig. 1).

### δ^18^O and δ^13^C composition

**Fig. 2.**
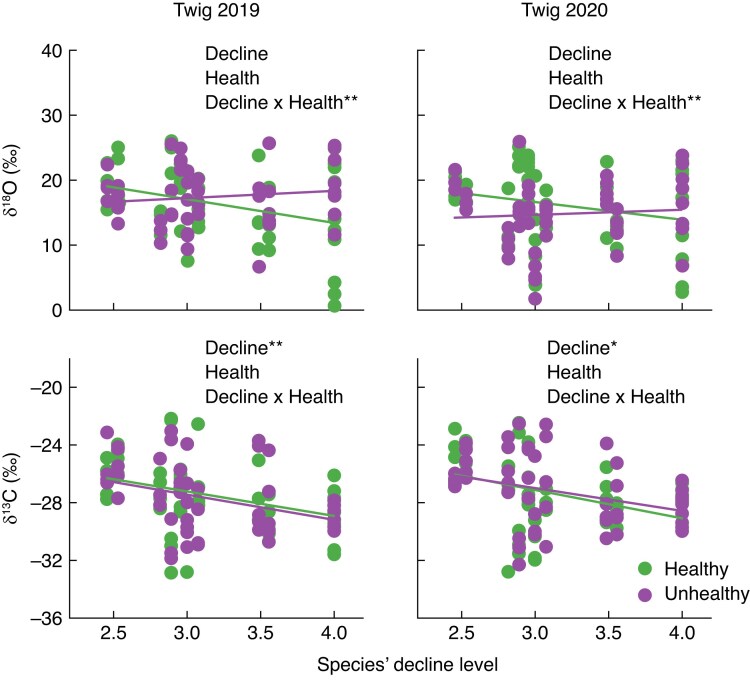
Isotopic composition (δ^18^O and δ^13^C) of twigs from 2019 and 2020 in healthy and unhealthy individuals of evergreen angiosperm species of the sclerophyllous Mediterranean forest of Central Chile with different decline level. Insets show statistical significance of decline, health status and their interaction; asterisks indicate statistically significant differences at *P < 0.05 and **P < 0.01.

The best models accounting for variation in δ^18^O included species decline, health status and the interaction of the two (Supplementary Data Table S1, Appendix S1). For δ^13^C, models including species decline, health status and their interaction had similar AIC as models with species decline only (Table S1, Appendix S1). Isotopic differences between healthy and unhealthy individuals were quite variable for the different species, particularly for δ^18^O (Fig. S1, Appendix S1). For δ^18^O, there was a significant interaction between the decline level and health status in both 2019 and 2020 twigs (Table 1). Specifically, in species with higher decline level, unhealthy individuals had higher δ^18^O (i.e. lower stomatal conductance) than healthy ones; conversely, in species with lower decline, healthy individuals had higher δ^18^O (i.e. lower stomatal conductance) than unhealthy ones (Fig. 2). δ^13^C decreased with increasing decline level in both 2019 and 2020 twigs (Table 1; Fig. 2), indicating a lower discrimination against ^13^C during C assimilation in species with lower decline. This pattern was similar for healthy and unhealthy individuals across the different decline levels (Table 1; Fig. 2).

**Fig. 3.**
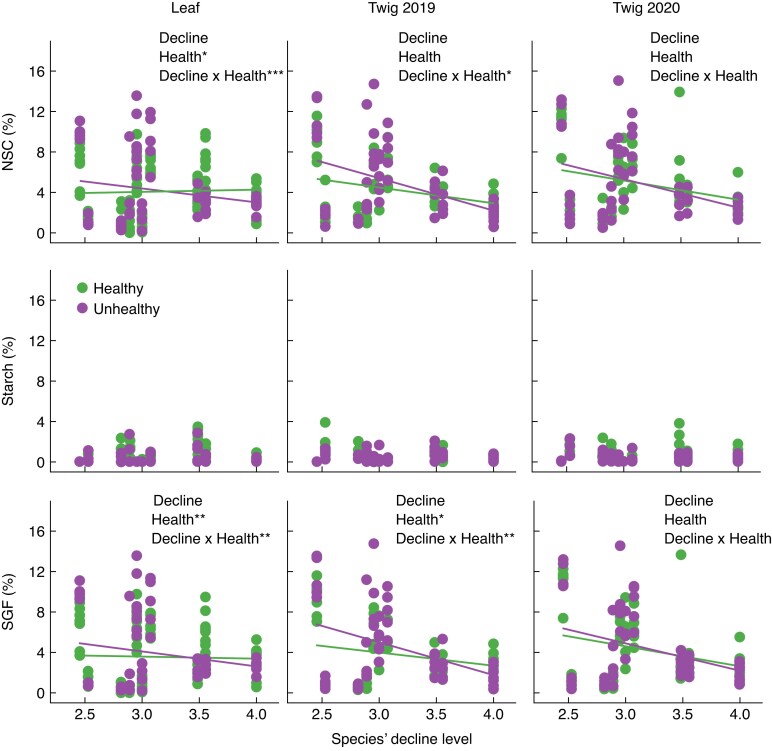
Concentrations of total non-structural carbohydrates (NSCs), starch and sugar (as a percentage of dry mass) of leaves and twigs (from 2019 and 2020) in healthy and unhealthy individuals of evergreen angiosperm species of the sclerophyllous Mediterranean forest of Central Chile with different decline level. Insets show statistical significance of decline, health status and their interaction; asterisks indicate statistically significant differences at *P < 0.05, **P < 0.01 and ***P < 0.001.

**Table 1.** Summary of F-ratios and inference (*P*-values) for the effects of species’ decline level, health status within species (healthy, unhealthy) and their interaction on the isotopic composition (δ^18^O and δ^13^C) of twigs formed in 2019 and 2020, and of species’ decline level, health status, age and their interactions on the mean basal area increment (BAI) for the periods 1999–2009 and 2010–2020 in angiosperm species of the sclerophyllous Mediterranean forest of Central Chile.

	d.f. (n, d)	F-ratio	*P*-value		d.f. (n, d)	F-ratio	*P*-value
δ^18^O_2019_				δ^13^C_2019_			
Intercept	1, 97	215.39	<0.001		1, 97	12 339.92	<0.001
Decline	1, 9	0.27	0.614		1, 9	12.10	0.007
Health status	1, 97	2.15	0.145		1, 97	0.097	0.727
Decline × Health status	1, 97	9.21	0.003		1, 97	0.286	0.972
δ^18^O_2020_				δ^13^C_2020_			
Intercept	1, 97	135.36	<0.001		1, 97	6681.38	<0.001
Decline	1,9	0.20	0.666		1, 9	7.161	0.025
Health status	1, 97	3.78	0.055		1, 97	0.216	0.643
Decline × Health status	1, 97	7.82	0.006		2, 97	0.185	0.668
BAI_1999–2009_				BAI_2010–2020_			
Intercept	1, 85	13.387	<0.001		1, 89	13.007	<0.001
Decline	1, 5	0.021	0.891		1, 5	0.015	0.906
Age	1, 85	0.864	0.355		1, 84	0.041	0.840
Health status	1, 85	24.491	<0.001		1, 89	17.67	<0.001
Decline × Health status	1, 85	0.344	0.559		1, 84	0.005	0.943
Decline × Age	1, 85	2.364	0.128		1, 84	3.863	0.053
Age × Health status	1, 85	0.052	0.819		1, 84	0.352	0.554
Decline × Health status × Age	1, 85	0.126	0.723		1, 84	0.352	0.554

d.f., degrees of freedom; n and d, numerator and denominator, respectively.

### NSC, starch and sugar concentrations

SGF was the main fraction of NSCs in leaves and twigs of all species and for both years, with starch concentrations being remarkable low (Fig. 3; Supplementary Data Fig. S2, Appendix S1). Only two species, *Lithraea* and *Myrceugenia*, had >1 % starch concentrations in twigs, and only one species (*Myrceugenia*) had >1 % starch concentrations in leaves (Fig. S2, Appendix S1).

The variation in NSCs and SGF was generally best predicted by the full models, i.e. including decline level, health status and their interaction. These LMMs indicated that leaf NSC and SGF concentrations, and SGF_twig_2019_ and NSC_twig_2019_, varied with the species’ decline level depending on the health status; unhealthy trees had lower concentrations than healthy ones at high levels of species-specific decline. Conversely, unhealthy trees had higher concentrations than healthy ones in species of low decline level (Table 2; Fig. 3). For the variation in starch of leaves, the models including the decline level (with or without interaction with health status) were not better than the random model (Table S1, Appendix S1). Variation in starch of twigs was best accounted for by models including the species decline only. However, these LMMs indicated that the decline level had no significant effect on starch concentrations in 2019 and 2020 twigs (Table 2).

**Table 2.** Summary of F-ratios and inference (*P*-values) for the effects of the species’ decline level, health status within species (healthy, unhealthy) and their interaction on the concentrations of total non-structural carbohydrates (NSCs), starch, and sucrose, glucose and fructose (SGF) of leaves (pooled for 2019 and 2020) and twigs formed in 2019 and 2020 in angiosperm species of the sclerophyll Mediterranean forest of Central Chile.

	d.f. (n, d)	F-ratio	*P*-value	d.f. (n, d)	F-ratio	*P*-value	d.f. (n, d)	F-ratio	*P*-value
	NSC_leaf_			NSC_twig_2019_			NSC_twig_2020_		
Intercept	1, 144	21.43	<0.001	1, 144	31.18	<0.001	1, 144	29.30	<0.001
Decline	1, 9	0.03	0.859	1, 9	1.86	0.206	1, 9	1.74	0.219
Health status	1, 144	4.83	0.029	1, 144	3.53	0.063	1, 144	0.35	0.557
Decline × Health status	1, 144	11.95	<0.001	1, 144	6.40	0.013	1, 144	3.48	0.065
	Starch_leaf_			Starch_twig_2019_			Starch_twig_2020_		
Intercept	1, 144	7.78	0.006	1, 144	13.07	<0.001	1, 144	14.57	<0.001
Decline				1, 9	0.19	0.673	1, 9	0.01	0.949
	SGF_leaf_			SGF_twig_2019_			SFG_twig_2020_		
Intercept	1, 144	15.22	<0.001	1, 144	19.73	<0.001	1, 144	19.08	<0.001
Decline	1, 9	0.10	0.760	1, 9	1.31	0.281	1, 9	1.40	0.266
Health status	1, 144	8.32	0.004	1, 144	4.32	0.040	1, 144	1.41	0.238
Decline × Health status	1, 144	10.03	0.002	1, 144	10.44	0.002	1, 144	3.41	0.068

d.f., degrees of freedom; n and d, numerator and denominator, respectively.

### Growth

**Fig. 4.**
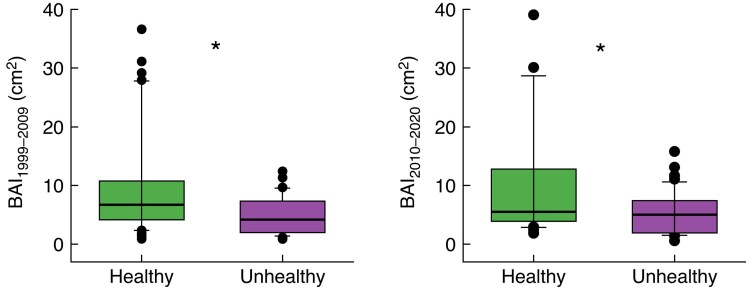
Mean basal area increment (BAI) for the periods 1999–2009 and 2010–2020 as a function of the tree health status (healthy, unhealthy) in species of the sclerophyllous Mediterranean forest of Central Chile in La Campana National Park. Asterisks indicate statistically significant differences between healthy and unhealthy trees (at *P < 0.05).

Mean BAI did not vary with the species-specific decline level, either during the pre-megadrought (1999–2009) or during the megadrought (2010–2020) period, and there was no significant interaction between decline and health status (Table 1). Healthy individuals had significantly higher BAI_1999–2009_ and BAI_2010–2020_ than unhealthy ones (Tables 1; Fig. 4). This result was mainly driven by four species (Supplementary Data Table S2, Fig. S3, Appendix S1). BAI increased with DBH, and more so in healthy than in unhealthy individuals, particularly for the period 1999–2009 (Table S3, Fig. S4, Appendix S1). In *Persea*, one of the most drought-sensitive species, healthy trees grew more than unhealthy trees during the pre-megadrought period, but this difference blurred during the megadrought (Table S2, Fig. S3, Appendix S1). In *Cryptocarya* and *Lithraea*, the two species with the lowest decline, growth was similar between healthy and unhealthy trees for both periods, with unhealthy trees actually increasing their BAI in 2020 relative to 2019 (Table S2, Fig. S3, Appendix S1).

## DISCUSSION

Contrary to our expectation, we did not find a generalized pattern of lower NSC concentrations in unhealthy trees when compared to healthy ones across 11 woody species exposed for >10 years to a megadrought in a site in Central Chile with sclerophyllous forest. Rather, we found that the magnitude and direction of the differences between healthy and unhealthy individuals depended on the level of drought-induced decline of the species, which was associated with biochemical and diffusive limitations to C assimilation. First, δ^13^C decreased with the species’ decline level in twigs formed before and during the hyperdrought (2019 and 2020, respectively) but δ^18^O did not, indicating that differences in C assimilation among the species studied are explained by biochemical limitations (Scheidegger *et al*., 2000), with lower C assimilation rates in the species with higher decline level. Second, in the less drought-sensitive species (i.e. lower decline level), healthy individuals showed lower stomatal conductance (i.e. higher δ^18^O) than unhealthy individuals, indicating a more timely and/or tighter stomatal control than in unhealthy individuals (Scheidegger *et al*., 2000). Although deeper root systems access water impoverished in ^18^O (Allison and Hughes, 1983), the differences in δ^18^O found in our study occurred at the intraspecific level and can hardly be attributed to potential interspecific variation in rooting depth. As healthy individuals were more frequent in species with a lower decline level, their response can be assumed to be a successful strategy to face drought and eventually to reduce the species-specific decline level. By contrast, in species with higher decline (i.e. high drought-sensitivity), δ^18^O was higher in unhealthy than in healthy individuals, indicating that most individuals of these species (which were unhealthy individuals, Table 1) experienced long-term stomatal closure. Given the low intrinsic rates of C assimilation of these species, prolonged stomatal closure led to a reliance on C reserves for metabolism, as indicated by the lower NSC and SGF concentrations in unhealthy than in healthy trees of highly drought-sensitive species (i.e. higher decline level). It is also possible that stomatal closure did not occur as much as anticipated so as to impede hydraulic failure and consequent generalized decline. Overall, these results point to intrinsic carboxylation capacity and timely stomatal control as critical processes in the resistance of these angiosperm woody species to prolonged drought.

Variability in biochemical and diffusive limitations on C assimilation is reflected in the NSC patterns. In the less drought-sensitive species, the lower SGF concentration in healthy individuals compared to unhealthy ones is consistent with the lower stomatal conductance (inferred through δ^18^O), which would be limiting photosynthesis. Accordingly, the metabolism of healthy individuals of these species was to some extent sustained by C reserves, as indicated by the lower levels of NSCs in the leaves and in the 2019 twigs in comparison to unhealthy individuals. Similarly, *Pinus edulis* significantly reduced sapwood starch concentrations after a decade of 45 % reduction in precipitation (Peltier *et al*., 2023). However, C reserves in the tissues examined were extremely low in all species, regardless of their drought-sensitivity and individual health status. This was particularly the case of starch concentration, which rarely exceeded 1 %, a value exceptionally low for angiosperms of Mediterranean regions (Piper *et al*., 2006; Rosas *et al*., 2013; Piper, 2015; Martínez-Vilalta *et al*., 2016; Palacio *et al*., 2018). Consistent with the frame of C starvation (McDowell *et al*., 2022), these results suggest that twig and leaf C reserves were depleted after many years of pervasive drought; they also point to a critical importance of intrinsic C assimilation rates as the ultimate mechanism sustaining dehydration tolerance. On the other hand, the higher NSC and SGF concentrations in unhealthy individuals of species with lower decline may reflect restrictions to sugar export caused by limited phloem translocation in dehydrated tissues (Sala *et al*., 2010; Sevanto *et al*., 2014), as these individuals had a high stomatal conductance and were evidently dehydrated. Dehydration in trees that maintained high stomatal conductance under drought points to hydraulic failure as a mechanism involved in the unhealthy status of trees belonging to species with lower decline (McDowell *et al*., 2022).

In the more drought-sensitive species, lower C assimilation rates (inferred from isotopic composition) in unhealthy individuals concurred with lower stomatal conductance. Consequently, unhealthy individuals had reduced NSC and SGF concentrations (leaf and twigs formed in 2019), which in turn appeared to limit the osmotic adjustment and dehydration tolerance (O’Brien *et al*., 2014). With depleted C reserves and low capacity to produce new sugars, the ability to cope with prolonged drought was reduced, thus explaining the high level of decline recorded for these species. Together, these results support C starvation as the causal mechanism of limited drought acclimation in the most drought-sensitive species (McDowell *et al*., 2022).

Unhealthy individuals grew less than healthy ones regardless of species-specific decline level, a finding that we did not expect. The fact that such differences were evident in the two periods evaluated suggests that factors other than the megadrought had affected these trees. In three of the seven species assessed for growth, we found that unhealthy individuals were smaller than healthy individuals, so the slower growth of unhealthy individuals could have been in part explained by their non-dominant position within the forest. However, the effect of health status on BAI was still highly significant when DBH was included in the model, which also showed that healthy individuals grew more than unhealthy ones in response to increasing DBH, particularly for the pre-megadrought period. It is possible that the effects of drought on the growth of these woody species may have started much earlier than the megadrought. Indeed, the megadrought is considered as an intensification, starting by 2010, of the water deficit that has been affecting the region since the end of the 20th century (Miranda *et al*., 2023). Our results are in line with findings reported for different species in other Mediterranean sites in Chile (Venegas-González *et al*., 2018; González-Reyes *et al*., 2024). On the other hand, although the decline level was not related to the growth rate, certain trends can be identified when the data are analysed species by species (Supplementary Data Table S1, Fig. S3, Appendix S1). The two species that showed similar growth between healthy and unhealthy trees during the two periods evaluated were those with the lowest decline level (*Lithraea* and *Cryptocarya*), with the BAI of both species being lower than most of the studied species (<10 cm^2^ in both healthy and unhealthy plants across the study period). In *Persea*, the species with the highest decline level, differences in growth were only detected before the megadrought, probably because healthy individuals became affected by the megadrought and adopted a similar growth pattern to that of unhealthy individuals. The fact that unhealthy individuals of the most drought-sensitive species not only grew less, but also had lower NSC concentrations and a low water use efficiency (i.e. high δ^18^O and low δ^13^C values), agrees with the framework of long-term C starvation driving a deterioration of the C balance and hydraulic performance (Cailleret *et al*., 2017). In contrast to the evidence shown by Cailleret *et al*. (2017), we found that long-term reduction in growth is also common in angiosperms. Xylogenesis, particularly cell-wall thickening, is a highly C-demanding process (Buttò *et al*., 2025), and probably more so in sclerophyllous species, which are generally characterized by a high wood density. The C requirements for wood formation in these species might have been limited, especially in unhealthy individuals where photosynthesis was strongly limited by reduced leaf area. The absence of a sudden growth reduction during the hyperdrought further supports the fact that C starvation is the process probably associated with tree decline in these species (Gessler *et al*., 2018).

Beyond biochemical and diffusive limitations to C assimilation, other functional traits could contribute to explaining interspecific differences in the drought-induced decline observed here. They might include root system depth (Nardini *et al*., 2016) or the ability to maintain cell turgor at low water content, a trait closely linked to sclerophylly (Bartlett *et al*., 2012). However, there is limited information about ecophysiological traits for the species included in our study, and even less for mature individuals under natural conditions. Consequently, the available information is insufficient to characterize other dimensions of the drought resistance strategies. For instance, the limited data on root systems reveal no clear pattern: while one of the most severely declining species has shallow roots (*Retanilla*, 0.71 m), species with the lowest decline level (*Lithraea*, 3.8–5 m; *Cryptocarya*, 1–2 m) have root systems as shallow as, or shallower, than species with intermediate decline level (*Quillaja*, 8 m; *Peumus*, 1.07 m) (Canadell and Zedler, 1995; Ayma Romay and Bown, 2021). Further studies are needed for a more comprehensive understanding of drought resistance strategies in woody plants of the Chilean Mediterranean.

## CONCLUSIONS

Stomatal control and osmotic adjustment are critical to drought resistance in Mediterranean ecosystems. While our results confirm the widely accepted role of C reserves supporting metabolism in the face of such diffusive limitations (e.g. McDowell *et al*., 2022), they also clearly show that the capacity to sustain metabolic demands ultimately depended on the intrinsic rates of C assimilation. Based on the analysis of carbon stable isotopes in tissues, the species with lower decline in Chile’s Mediterranean sclerophyllous forests exhibit a greater carboxylation capacity and tighter stomatal control, which prevents dehydration. Conversely, tight stomatal regulation was associated with dehydration when it did not come along with high intrinsic capacity of C assimilation. The maintenance of high C assimilation rates after a long-lasting severe drought was therefore identified as a predictor of decline in woody species from a Mediterranean biome.

This finding opens possibilities for forest restoration and conservation. The search for drought-resistant species appears urgent under climatic projections anticipating continued or exacerbated hot and dry conditions for Mediterranean areas. Policies aiming to conserve (or restore with) species with high intrinsic capacity of C assimilation emerge as the most viable strategy for climate change mitigation in these forests. Information on intrinsic capacity of C assimilation can be obtained through isotopic analysis and, in some cases, it can be also inferred through other approaches of diverse complexity (tissue nitrogen concentration, gas exchange analysis, enzymatic activity, etc.). Mitigation of climate change via C sequestration through vegetal biomass is therefore associated with the capacity to resist drought. Conversely, the species with lower decline exhibited low growth rates, emphasizing that growth rate is not an adequate attribute to target restoration and conservation, and eventually C sequestration, in these forests.

## Supplementary Material

mcag100_Supplementary_Data

## SUPPLEMENTARY DATA

Supplementary data are available at Annals of Botany  *online* and consist of the following. Table S1: Models predicting the isotopic composition (δ^18^O, δ^13^C) and concentration of non-structural carbohydrates (NSC), starch and sugars (sucrose, fructose and glucose, SFG) in healthy and unhealthy individuals of species with different decline level in Central Chile. Table S2: Number of healthy and unhealthy individuals and the *P*-values of comparisons between the mean basal area increment of healthy and unhealthy trees for the 1999–2009 (pre-megadrought) and 2010–2020 (megadrought) periods. Table S3: Summary of F-ratios and inference (*P*-values) for the effects of species’ decline level, tree size (as diameter at breast height, DBH), health status within species (healthy, unhealthy) and their interactions on the mean basal area increment (BAI) for the periods 1999–2009 and 2010–2020 in angiosperm species of the sclerophyllous Mediterranean forest of Central Chile. Figure S1: Oxygen and carbon isotopic composition (δ^18^O and δ^13^C) in twigs formed in 2019 and 2020, in healthy and unhealthy individuals of species with different decline level in Central Chile. Figure S2: NSCs, starch and SGF (%) in leaf and in twig segments formed in 2019 and 2020, in healthy and unhealthy individuals of species with different decline level in Central Chile. Figure S3: Annual basal area increment (BAI, in cm^2^) from 1999 to 2020 of healthy and unhealthy individuals of seven tree species with different decline level in Central Chile. Figure S4: Mean basal area increment (BAI) for the period 1999–2009 as a function of the diameter at breast height (DBH, in cm) of trees belonging to two categories of health status (healthy, unhealthy) in seven species of the sclerophyllous Mediterranean forest of Central Chile in La Campana National Park.
