# Supplementary material for: Tight stomatal regulation and high intrinsic capacity of carbon assimilation inferred from isotopic composition negatively correlate with drought-driven decline in sclerophyllous species
Source: Ann Bot. 2026 Apr 24;138(2):536–47. doi: 10.1093/aob/mcag100 (PMC13454689; doi:10.1093/aob/mcag100)

Supplementary Data

Tight stomatal regulation and high intrinsic capacity of carbon assimilation inferred from isotopic composition negatively correlate with drought-driven decline in sclerophyllous species

Table S1. Models predicting the isotopic composition ($\delta^{18}O$, $\delta^{13}C$) and concentration of total non-structural carbohydrates (NSC), starch and sugars (sucrose, fructose and glucose, SFG) in leaf and twig (formed in 2019 or 2020) of healthy and unhealthy individuals of evergreen woody species with different decline level in La Campana National Park, Chile. AICc = Akaike Information Criterion corrected for small sample size. dec stands for decline level; trat stands for health status.

Response Variable	Models	AICc
$\delta^{18}O_{twig\_2019}$	δ^{18}O ~ 0,random=~1|sp,data=data_2019	684.81
	δ^{18}O ~ dec,random=~1|sp,data=data_2019	648.15
	δ^{18}O ~ dec * trat,random=~1|sp,data=data_2019	637.33
$\delta^{18}O_{twig\_2020}$	δ^{18}O ~ 0,random=~1|sp,data=data_2020	645.55
	δ^{18}O ~ dec,random=~1|sp,data=data_2020	613.11
	δ^{18}O ~ dec * trat,random=~1|sp,data=data_2020	602.77
$\delta^{13}C_{twig\_2019}$	δ^{13}C ~ 0,random=~1|sp,data=data_2019	565.18
	δ^{13}C ~ dec,random=~1|sp,data=data_2019	490.98
	δ^{13}C ~ dec * trat,random=~1|sp,data=data_2019	493.42
$\delta^{13}C_{twig\_2020}$	δ^{13}C ~ 0,random=~1|sp,data=data_2020	568.12
	δ^{13}C ~ dec,random=~1|sp,data=data_2020	498.92
	δ^{13}C ~ dec * trat,random=~1|sp,data=data_2020	500.95
NSC_{leaves}	NSC ~ 0,random=~1|sp,data=leaf	682.50
	NSC ~ dec,random=~1|sp,data=leaf	668.70
	NSC ~ dec * trat,random=~1|sp,data=leaf	656.32
$Starch_{leaves}$	Starch ~ 0,random=~1|sp,data=leaf	295.39
	Starch ~ dec,random=~1|sp,data=leaf	294.75
	Starch ~ dec * trat,random=~1|sp,data=leaf	298.12
SGF_{leaves}	SGF ~ 0,random=~1|sp,data=leaf	667.90
	SGF ~ dec,random=~1|sp,data=leaf	656.35
	SGF ~ dec * trat,random=~1|sp,data=leaf	642.78
NSC_{twig_2019}	NSC ~ 0,random=~1|sp,data=twig_2019	519.51
	NSC ~ dec,random=~1|sp,data= twig_2019	502.57
	NSC ~ dec * trat,random=~1|sp,data= twig_2019	496.05

Starch$_{twig_2019}$	Starch ~ 0,random=~1|sp,data=twig_2019	190.35
	Starch ~ dec,random=~1|sp,data= twig_2019	187.78
	Starch ~ dec * trat,random=~1|sp,data= twig_2019	193.24
SGF$_{twig_2019}$	SGF ~ 0,random=~1|sp,data=twig_2019	506.54
	SGF ~ dec,random=~1|sp,data= twig_2019	492.81
	SGF ~ dec * trat,random=~1|sp,data= twig_2019	482.23
NSC$_{twig_2020}$	NSC ~ 0,random=~1|sp,data=twig_2020	517.91
	NSC ~ dec,random=~1|sp,data= twig_2020	501.00
	NSC ~ dec * trat,random=~1|sp,data= twig_2020	500.12
Starch$_{twig_2020}$	Starch ~ 0,random=~1|sp,data=twig_2020	214.44
	Starch ~ dec,random=~1|sp,data= twig_2020	210.98
	Starch ~ dec * trat,random=~1|sp,data= twig_2020	214.93
SGF$_{twig_2020}$	SGF ~ 0,random=~1|sp,data=twig_2020	513.64
	SGF ~ dec,random=~1|sp,data= twig_2020	499.68
	SGF ~ dec * trat,random=~1|sp,data= twig_2020	497.92

Table S2. Number of healthy and unhealthy individuals that are part of the tree-ring chronologies per species and the *P-values* of comparisons between the mean basal area increment of healthy and unhealthy individuals, according to Wilcoxon rank sum test. *P-values* are shown for the 1999-2009 (pre-megadrought) and 2010-2020 (megadrought) periods. *P-values* of diameter at breast height (DBH) comparisons between healthy and unhealthy individuals, according to t-tests are also shown.

Species	$n_{healthy, unhealthy}$	$DBH_{healthy, unhealthy}$	*P-value* DBH_{u-h}	*P-value* $BAI_{1999-2009\ h-u}$	*P-value* $BAI_{2010-2020\ h-u}$
Schinus latifolius	6, 7	21.4 ± 1.8, 13.5 ± 1.5	0.007	< 0.001	< 0.001
Quillaja saponaria	8, 8	34.1 ± 2.6, 20.1 ± 1.0	0.0002	< 0.001	< 0.001
Cryptocarya alba	8, 7	20.7 ± 1.6, 18.9 ± 1.5	0.24	0.133	0.116
Peumus boldus	8, 7	16.9 ± 1.6, 9.4 ± 0.7	0.001	< 0.001	< 0.001
Persea lingue	10, 7	20.0 ± 2.4, 18.9 ±3.7	0.75	< 0.001	0.193
Lithraea caustica	9, 7	15.6 ± 1.6, 14.3 ± 2.1	0.65	0.699	0.076
Azara celastrina	7, 8	18.3 ± 2.6, 11.6 ± 1.5	0.09	< 0.001	< 0.001

n stands for the number of individuals sampled for each category of health status; DBH stands for diameter at breast height (mean ± standard error)

Table S3. Summary of F-ratios and inference (P-values) for the effects of species' decline level, tree size (as diameter at breast height, DBH), health status within species (healthy, unhealthy), and their interactions, on the mean basal area increment (BAI) for the periods 1999-2009 and 2010-2020 in angiosperm species of the sclerophyllous Mediterranean forest of Central Chile.

	Df (n, d)	F-ratio	P-value	Df (n, d)	F-ratio	P-value
	$BAI_{1999-2009}$			$BAI_{2010-2020}$		
Intercept	1, 87	67.837	<0.001	1, 89	28.490	<0.001
Decline	1, 5	0.105	0.761	1, 5	0.037	0.855
DBH	1, 87	145.540	<0.001	1, 84	31.345	<0.001
Health Status	1, 87	67.531	<0.001	1, 89	22.474	<0.001
Decline × Health Status	1, 87	2.874	0.094	1, 84	0.409	0.524
Decline × DBH	1, 87	0.233	0.631	1, 84	2.331	0.130
DBH × Health Status	1, 87	35.090	<0.001	1, 84	3.997	0.049
Decline × Health Status × DBH	1, 87	1.103	0.296	1, 84	0.051	0.822

Df stands for degree of freedom; n and d stand for numerator and denominator, respectively

Figure S1. Carbon and oxygen isotopic composition in healthy and unhealthy trees of eleven species exhibiting different decline level in La Campana National Park, Chile. Species are referred to by their genus and ordered from the lowest decline level (left) to the highest decline level (right); for complete species names and details on their decline level please see Table 2. Boxes delimit the interquartile range between quartile 1 (Q1, 25%) and Q2 (75%); the mean line within the box stands for the median (Q2); the cross shows the mean; whiskers show the data's range outside the interquartile range (IQR), i.e. 1.5 times the IQR below and above. Points below or above whiskers show outliers, i.e. data that fall below the first quartile (Q1) minus 1.5 times the interquartile range (IQR), or above the third quartile (Q3) plus 1.5 times the IQR.

33

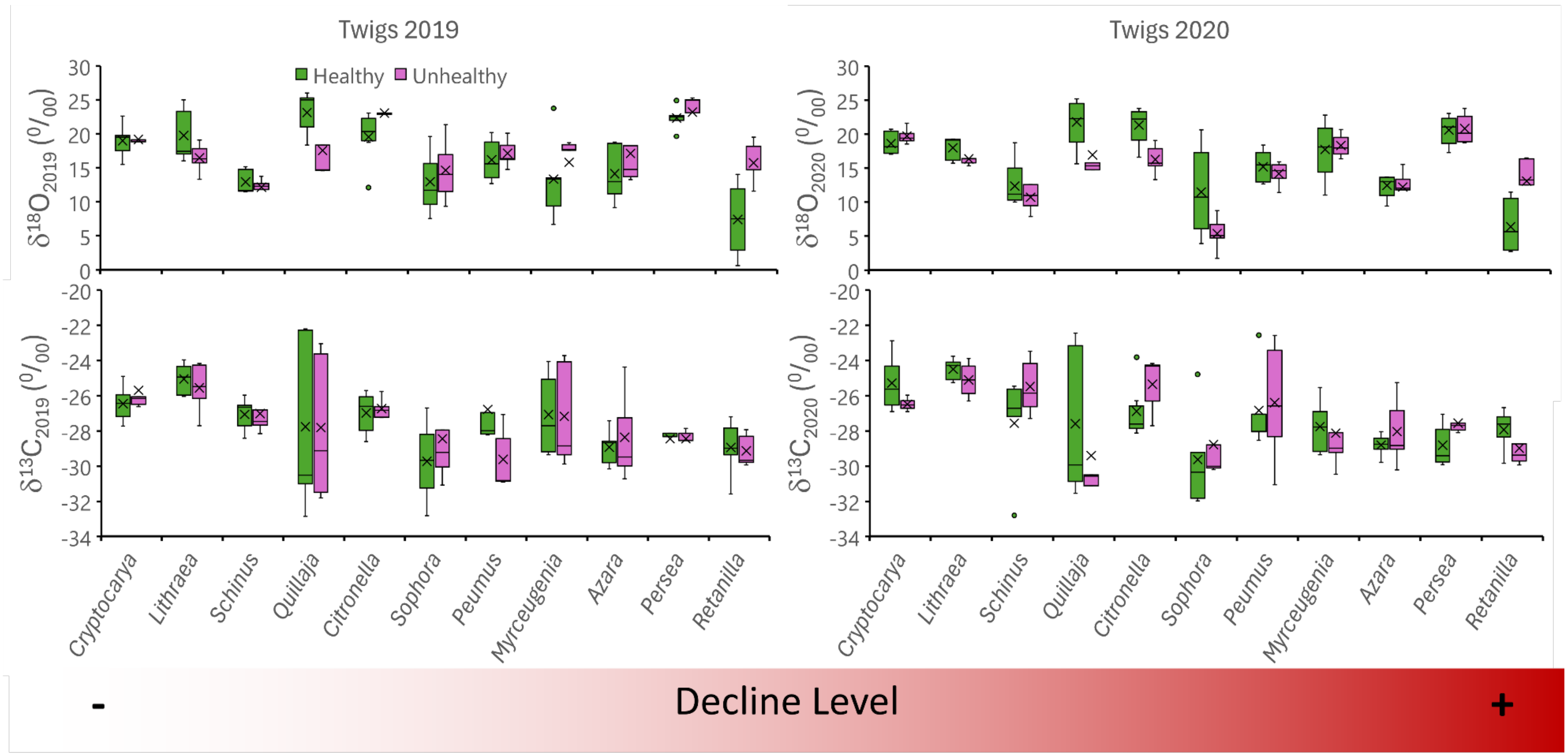

Twigs 2019
Twigs 2020
Healthy
Unhealthy
$\delta^{18}O_{2019}$ (0/00)
$\delta^{13}C_{2019}$ (0/00)
$\delta^{18}O_{2020}$ (0/00)
$\delta^{13}C_{2020}$ (0/00)
Cryptocarya
Lithraea
Schinus
Quillaja
Citronella
Sophora
Peumus
Myrceugenia
Azara
Persea
Retanilla
Decline Level
-
+

Figure S2. Total NSC, starch and SGF (%) in leaf and in twig segments formed in 2019 and 2020, in healthy and unhealthy individuals of evergreen species exhibiting different decline level in La Campana National Park, Chile. Species are referred to by their genus and ordered from the lowest decline level (left) to the highest decline level (right); for complete species names and details on their decline level please see Table 2. Boxes delimit the interquartile range between quartile 1 (Q1, 25%) and Q2 (75%); the mean line within the box stands for the median (Q2); the cross shows the mean; whiskers show the data's range outside the interquartile range (IQR), i.e. 1.5 times the IQR below and above. Points below or above whiskers show outliers, i.e. data that fall below the first quartile (Q1) minus 1.5 times the interquartile range (IQR), or above the third quartile (Q3) plus 1.5 times the IQR.

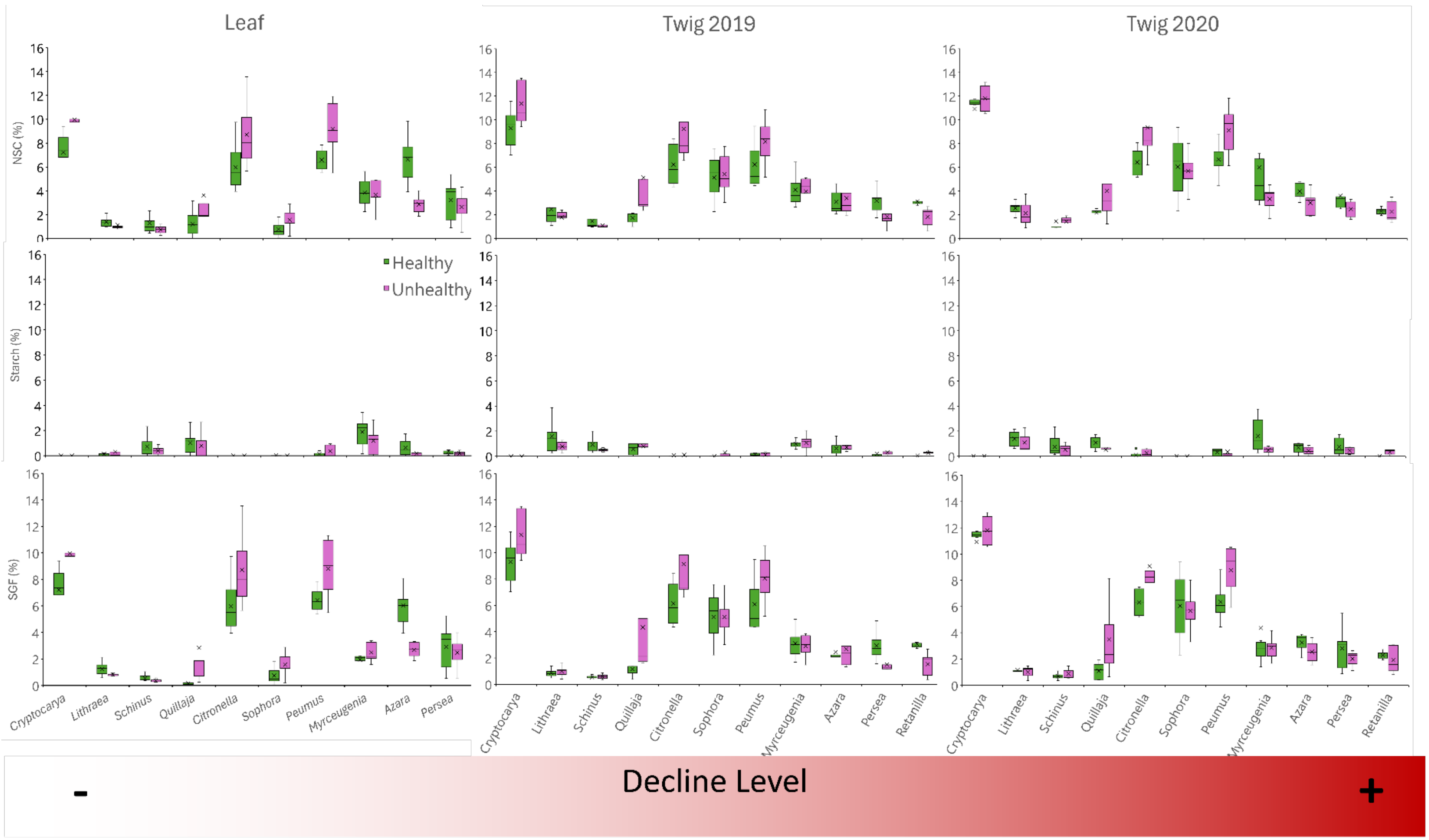
Leaf
Twig 2019
Twig 2020
NSC (%)
Starch (%)
SGF (%)
Healthy
Unhealthy
Cryptocarya
Lithraea
Schinus
Quillaja
Citronella
Sophora
Peumus
Myrceugenia
Azara
Persea
Retanilla
-
Decline Level
+

43

Figure S3. Annual basal area increment (BAI, in cm^2) from 1999 to 2020 of healthy and unhealthy individuals of evergreen tree species with different decline level in Central Chile. Solid lines show the mean; stripes show ± 1 standard error of the mean (n as in Table S1). Species are ordered from the lowest decline level (left) to the highest decline level (right).

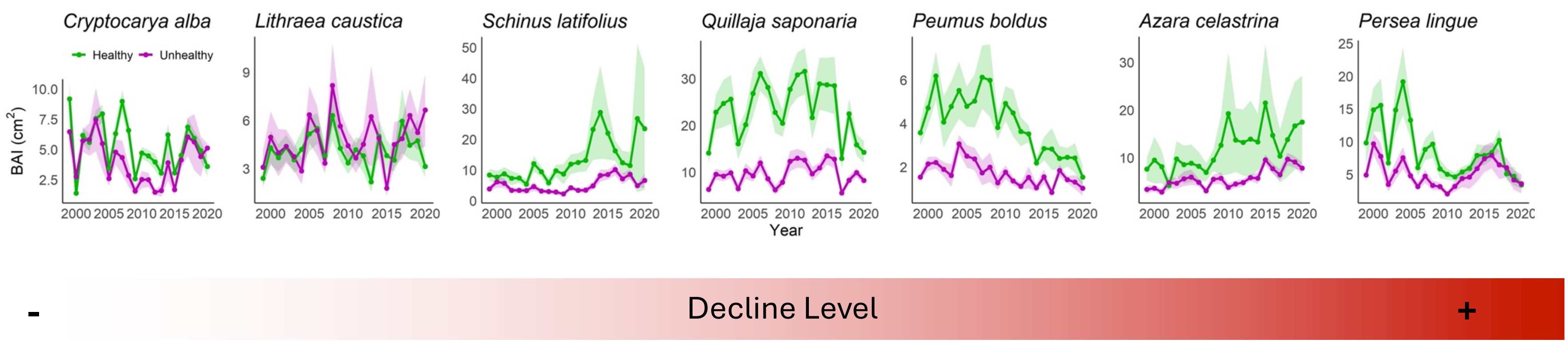

Figure S4. Mean basal area increment (BAI) for the period 1999-2009 as a function of the diameter at breast height (DBH, in cm) of trees belonging to two categories of health status (healthy, unhealthy) in seven species of the sclerophyllous Mediterranean forest of Central Chile in La Campana National Park.

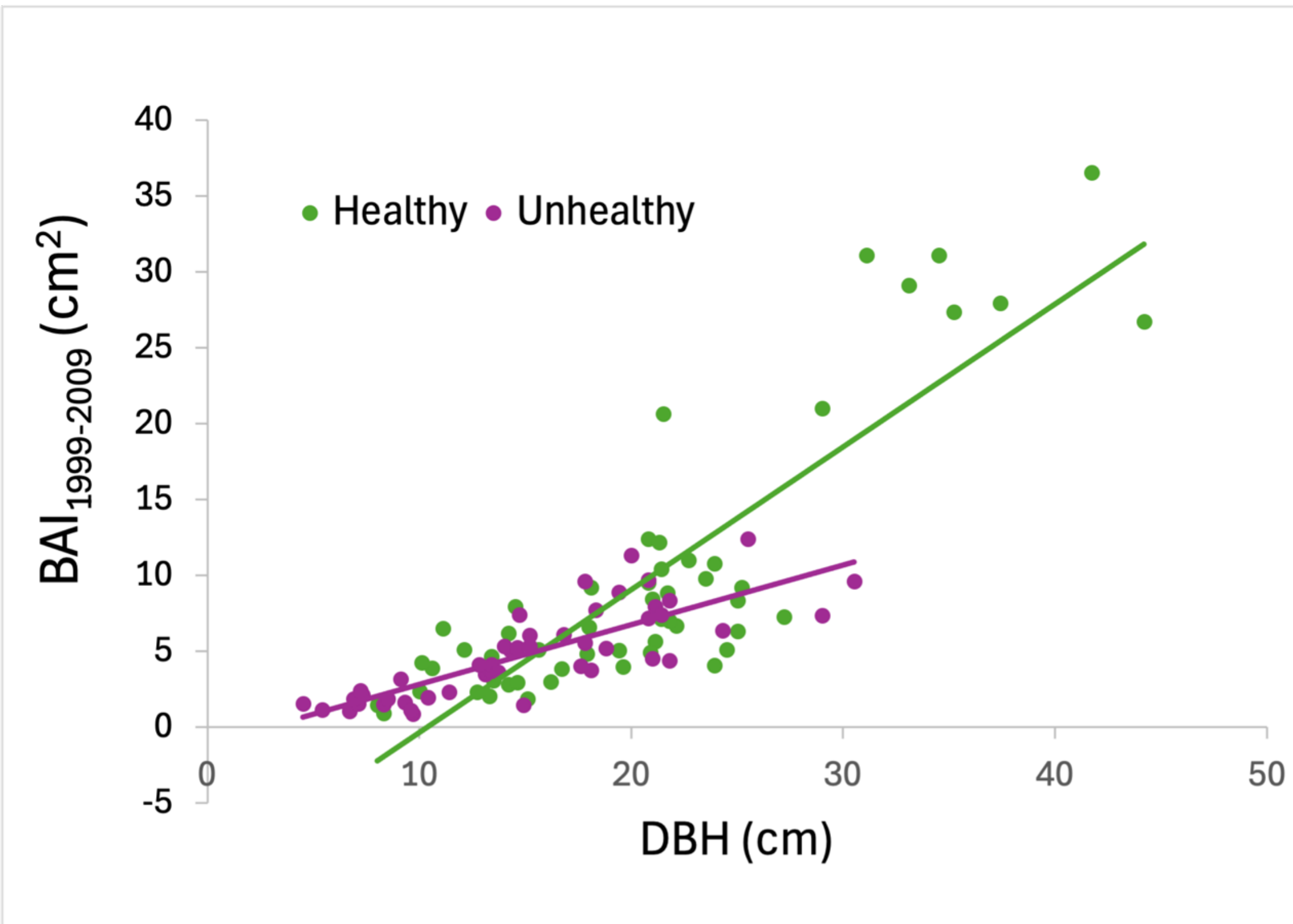

Supplement: Supplementary file 1 [file mcag100_supplementary_data.pdf]
